# Hydrophilic Partitioning or Surface Adsorption? A Quantitative Assessment of Retention Mechanisms for Hydrophilic Interaction Chromatography (HILIC)

**DOI:** 10.3390/molecules28186459

**Published:** 2023-09-06

**Authors:** Yong Guo, Dominik Baran

**Affiliations:** School of Pharmacy and Health Sciences, Fairleigh Dickinson University, Florham Park, NJ 07932, USA

**Keywords:** retention mechanism, partitioning, adsorption, phase ratio, quantitative, HILIC

## Abstract

Retention mechanisms in HILIC have been investigated and reported in literature. However, the current understanding of retention mechanisms is qualitative and lacks quantitative details. Previously, mechanism elucidation was based on indirect evidence, and unambiguous assignment of retention mechanisms has not been reported based on direct data. This study aims to quantitatively determine the contributions of two major retention mechanisms in HILIC, hydrophilic partitioning and surface adsorption to the overall retention of neutral compounds. Using the methodologies we developed previously, the phase ratio for adsorbed water layer and distribution coefficients were measured and used to calculate the retention factors contributed by hydrophilic partitioning. The methodology allows the determination of the contribution of surface adsorption simultaneously. The evaluation of five test compounds demonstrates that the retention may be controlled by hydrophilic partitioning, surface adsorption or both depending on compound characteristics. Quantitative assessment of retention mechanisms also makes it possible to better understand the effect of acetonitrile on retention in HILIC.

## 1. Introduction

Hydrophilic interaction chromatography (HILIC) has established itself as a unique mode of chromatographic separation over the past three decades. Often considered complementary to reversed-phase liquid chromatography (RPLC), HILIC provides stronger retention to small polar compounds on polar stationary phases using a mobile phase of water and organic solvents (e.g., acetonitrile) [1,2,3,4,5,6]. In his seminal paper published in 1990, Alpert postulated the retention mechanisms for HILIC. Since then, there have been continuous efforts in investigating and elucidating the retention mechanisms [7,8,9,10,11,12,13,14]. Published studies indicate that hydrophilic partitioning, surface adsorption, and electrostatic interactions may all play significant roles in retaining polar compounds in HILIC. Small polar compounds partition between the aqueous organic mobile phase and an immobilized water layer on the surface of polar stationary phases. The retention due to hydrophilic partitioning depends on the polarity of analytes (i.e., distribution coefficient) and the thickness of the adsorbed water layer [14]. Surface adsorption is often used to describe direct interactions between polar compounds and stationary phases through specific polar interactions (e.g., hydrogen bonding and polar-polar interactions) or non-specific van der Waals forces. A recent NMR study has shown that small polar compounds can interact directly with the ligands of the stationary phase [15]. If both polar compounds and stationary phases are charged, electrostatic interactions also influence retention, either enhancing retention through attractive interactions or reducing retention through repulsive interactions [16,17].

In RPLC, partitioning is considered the predominant retention mechanism, and adsorption and electrostatic interactions are deemed secondary interactions [18,19,20]. In comparison, the retention mechanisms in HILIC are more complex and less well understood. In a review article published in 2006, Hemstrom and Irgum attempted to evaluate the retention mechanisms by fitting existing retention data reported in literature with either a partitioning model (Equation (1)) or an adsorption model (Equation (2)) [21]:

(1) $$logk^{\prime }=logk_{o}^{\prime }-S\varphi$$

(2) $${logk}^{\prime }={logk}_{B}^{\prime }-mlogN_{B}$$

where *φ* and *N_B_* represent the volume fraction and the mole fraction of the stronger solvent (water) in the mobile phase, and $k_{o}^{\prime }$ and $k_{B}^{\prime }$ are hypothetical retention factors when *φ* = 0 or *N_B_* = 1. Neither model was shown able to fit all the retention data, which led to the question whether HILIC was based on partitioning or adsorption [21]. A mixed-model equation essentially combining Equations (1) and (2) was proposed by Liang and co-workers [22]:

(3) $${logk}^{\prime }=a+b\varphi +clog\varphi$$

The retention data of multiple compounds on various stationary phases was shown to fit the mixed-model equation better than either the partitioning model (Equation (1)) or the adsorption model (Equation (2)). It implies that both partitioning and adsorption contribute to the retention in HILIC. However, Equation (3) does not provide any information on how much each mechanism contributes to the overall retention of test compounds, thus cannot answer the question, what is the predominant retention mechanism in a given chromatographic system (column and mobile phase).

When multiple mechanisms are involved in a chromatographic process, the overall retention is the sum of all the contributions of individual mechanisms. If individual contribution of each retention mechanism could be quantitatively determined, it would significantly improve our understanding of the retention process. Unfortunately, determining contributions of various retention mechanisms has never been reported for RPLC or HILIC. This is likely because there was not a suitable methodology available for this purpose. We recently developed a methodology to determine the contributions of the hydrophilic partitioning and surface adsorption mechanisms to the observed retention of non-ionized compounds in HILIC [23]. The methodology is premised on the thermodynamic concept that the retention factor is the product of distribution coefficient and phase ratio in a partitioning-driving process [24]. If the distribution coefficient is constant in the mobile phase, the retention factor should be linearly proportional to the phase ratio. The challenge in varying the phase ratio is solved by changing the salt concentration in the mobile phase at a fixed acetonitrile level. Although the methodology has been demonstrated to be valid on different stationary phases, only one model compound (cytosine) was used in the published study. Obviously, the methodology needs to be applied to more compounds with different behaviors in HILIC.

**Figure 1:**
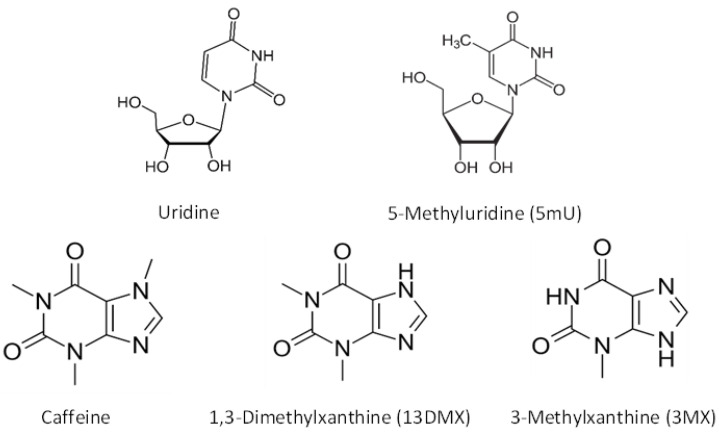
Structures of the selected test compounds.

In the current study, five probe compounds were selected to evaluate the retention mechanisms in HILIC. Uridine and its derivative, 5-methyluridine (structures shown in Figure 1) were included in the test mixture because they are used to probe methylene selectivity of various polar stationary phases [25]. In addition, caffeine and its two demethylated metabolites, namely, 1,3-dimethylxanthine (theophylline) and 3-xanthine, were added to the mixture since they were used to investigate the retention mechanisms in a previous study [10]. The working hypothesis for this study was that the retention of the five selected compounds was controlled by both hydrophilic partitioning and surface adsorption. The retention of the selected probe compounds was evaluated mainly on a polyacrylamide stationary phase and in different mobile phase conditions. The study results clearly demonstrate that quantitative information on retention contribution can provide more insights into the retention mechanisms in HILIC and helps answer the question whether HILIC is based on partitioning or adsorption.

## 2. Results and Discussion

In a partitioning-driven separation process, the retention is controlled by both distribution coefficient (*K*) and phase ratio (∅):

(4) $$k_{par}^{\prime }=K\emptyset$$

The adsorbed water on the packing surface can be considered *de facto* stationary phase for the partitioning process in HILIC. Therefore, Equation (4) can be used to calculate the retention factor contributed by hydrophilic partitioning if both the distribution coefficient and the phase ratios are available. For a neutral compound without any electrostatic interactions with the stationary phase, it is hypothesized that the retention factor is linearly proportional to the phase ratio. The overall retention is expressed as the sum of the contributions from both hydrophilic partitioning and surface adsorption:

(5) $$k^{\prime }=k_{ads}^{\prime }+K\emptyset$$

where $k_{ads}^{\prime }$ is the retention factor contributed by surface adsorption. It is assumed that in a mobile phase with a fixed level of acetonitrile, the distribution coefficient and surface adsorption are not affected by the salt concentration in the range typically used in HILIC. The validity of this assumption was discussed, and the hypothesis has been demonstrated to be true in our previous publication [23]. Based on Equation (5), the slope of the linear regression line provides the distribution coefficient, and the intercept indicates the retention factor contributed by surface adsorption.

### 2.1. Phase Ratio

**Figure 2:**
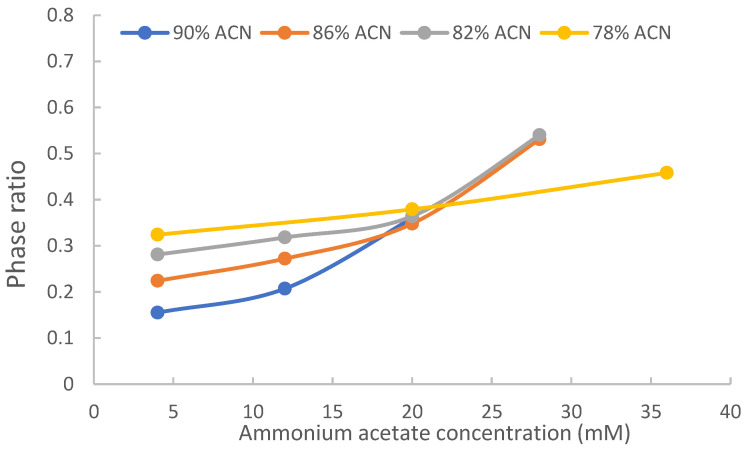
Variation of phase ratio with acetonitrile level and ammonium acetate concentration on the TSKgel Amide 80 column at 25 °C.

In this study, toluene was chosen as the void marker to measure the phase ratio using the method that we developed previously [26]. McCalley’s work has shown that the use of toluene as the void marker provides acceptable accuracy for measuring the volume of the adsorbed water layer [27]. Figure 2 shows the measured phase ratio of TSKgel Amide-80 column (polyacrylamide phase) at various acetonitrile and salt levels. Consistent with previous reports, the current data indicates that the phase ratio varies with both acetonitrile level and salt concentration in the mobile phase. First, the phase ratio increases with the salt concentration at all acetonitrile levels due to increasing volume of the adsorbed water layer as the salt concentration increases. Second, the phase ratio is smaller at higher acetonitrile levels and lower salt concentrations (<10 mM). Higher salt concentrations raise the phase ratio significantly in the mobile phase containing high acetonitrile levels. On the TSKgel Amide-80 column, the phase ratio is similar at 20 mM ammonium acetate concentration when the acetonitrile level changes from 90% to 82% and is also very close at 28 mM ammonium acetate concentration between 82% and 86% acetonitrile. At 78% acetonitrile, the phase ratio at 36 mM ammonium acetate is lower than that at 20 mM in 82% or 86% acetonitrile. These results are consistent with previous findings on XBridge Amide, LUNA HILIC and ZIC-HILIC columns and have significant implications on the retention in HILIC [23].

### 2.2. Quantitative Retention Assessment

**Figure 3:**
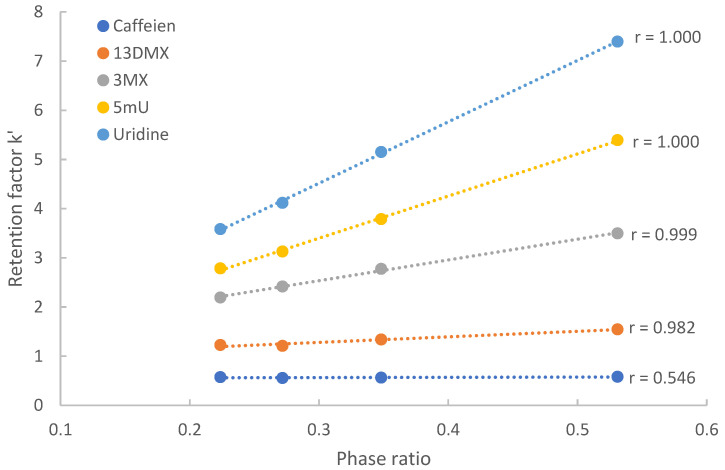
Plots of retention factor *k*′ vs. phase ratio (∅) for five selected test compounds on the TSKgel Amide 80 column at 25 °C.

The distribution coefficients of polar compounds between the aqueous organic mobile phase and the adsorbed water layer were determined using the method that we previously reported [23]. The test compounds were separated using the mobile phase with a fixed acetonitrile level but increasing salt concentration. Figure 3 shows the plot of the retention factors of five test compounds vs. the phase ratio in the mobile phase containing 86% acetonitrile. Linear regression demonstrates that the retention factors are linearly proportional to the phase ratio with high correlation coefficients (r > 0.98) for uridine, 5-methyluridine, 3-methylxanthine and 1,3-dimethylxanthine. The retention factors of caffeine are not significantly affected by the phase ratio, as indicated by low correlation coefficient (r = 0.546).

The slopes of the regression lines provide the distribution coefficients, and the intercepts represent the retention factors contributed by surface adsorption. Using the phase ratio and distribution coefficient data, the retention factors contributed by hydrophilic partitioning were calculated using Equation (4). Table 1 presents the distribution coefficients, calculated retention factors by partitioning ($k_{par}^{\prime }$), and the retention factors by surface adsorption ($k_{ads}^{\prime }$) on the TSKgel Amide-80 column in the mobile phase containing 90% acetonitrile and 20 mM ammonium acetate, which was the mobile phase condition that resulted in the strongest retention due to both large distribution coefficients at 90% acetonitrile level and a relatively high phase ratio at 20 mM ammonium acetate (Figure 2). The percentage numbers in parentheses represent the relative contributions of partitioning and adsorption and add up to close to 100% for uridine, 5-methyluridine, 3-methylxanthine and 1,3-dimethylxanthine.

As shown in Table 1, hydrophilic partitioning contributes approximately 91% and 80% of the overall retention for uridine and 5-methyluridine, respectively. This clearly demonstrates that hydrophilic partitioning is the predominant retention mechanism for uridine and 5-methyluridine. In comparison, the contribution of surface adsorption is relatively small, indicating surface adsorption is a minor mechanism particularly for uridine. On the other end of the spectrum, caffeine’s retention is entirely controlled by surface adsorption since its retention factor virtually does not vary with the phase ratio with low correlation coefficient (r = 0.546). Surface adsorption is also the predominant retention mechanism for 1,3-dimethylxanthine, contributing over 80% to the overall retention. For 3-methylxanthine, the data suggests that both partitioning and adsorption are significant retention mechanisms. It is interesting to note that for three methylated xanthine derivatives including caffeine (1,3,7-trimethylxanthine), the distribution coefficients decrease significantly with the addition of each methyl group (Figure 1) due to increased lipophilicity. This results in a drastic decrease in partitioning-controlled retention. In addition, the surface adsorption also seems to weaken for the xanthine derivatives with more methyl groups ($k_{ads}^{\prime }$). Both factors contribute to lower retention factors of caffeine and theophylline on the TSKgel Amide-80 column.

The retention of uridine and 5-methyluridine was evaluated on two polyhydroxy phases (EPIC HILIC and YMC PVA columns) in the mobile phase containing 85% acetonitrile with the ammonium acetate concentration ranging from 5–30 mM. Table 2 presents the phase ratio, distribution coefficients, and intercepts from linear regression for uridine and 5-methyluridine on EPIC HILIC and YMC PVA columns. The two columns have similar chemistry and phase ratios, and the distribution coefficients obtained by linear regression of *k*′ vs. ∅ data are not significantly different based on t-test. Surprisingly, the intercepts for uridine show small, but significant negative values. Since the intercept represents the retention attributed to surface absorption, the intercept may be small, but should be positive. In addition, the retention factors calculated for partitioning (*K*∅) are larger than the actual retention factors (~116%). This difference cannot be attributed to experimental errors.

A possible explanation of the negative intercept might be related to the phase ratio, which is assumed to indicate the volume of the adsorbed water layer. Equation (5) assumes that the entire adsorbed water is accessible to partitioning of polar compounds. However, this assumption may not be valid if a portion of the adsorbed water layer is not assessable, thus not involved in the partitioning process. The inaccessible water effectively reduces the phase ratio. If ∅′ indicate the portion of the phase ratio not available for partitioning, Equation (5) can be modified to Equation (6), which is rearranged to Equation (7).

(6) $$k^{\prime }=k_{ads}^{\prime }+K(\emptyset -\emptyset^{\prime })$$

(7) $$k^{\prime }={(k}_{ads}^{\prime }-{K\emptyset }^{\prime })+K\emptyset$$

In the above scenario, the distribution coefficients are not affected; therefore, Equation (7) still indicates that the retention factor is linearly proportional to the phase ratio. However, the intercept would be the retention factor from adsorption ($k_{ads}^{\prime }$) minus a small quantity represented by *K*∅′. If *K*∅′ ≥ $k_{ads}^{\prime }$, this would result in zero or negative intercepts. This might explain the negative intercept for uridine and close to zero intercept for 5-methyluridine. In this case, the contribution by surface adsorption cannot be accurately determined without knowing the ∅′ value. This phenomenon was found on two polyhydroxy stationary phases (EPIC HILIC and YMC PVA columns), but not on the polyacrylamide phase (TSKgel Amide-80 column).

### 2.3. The Effect of Acetonitrile Content

#### 2.3.1. The Effect on Hydrophilic Partitioning

**Figure 4:**
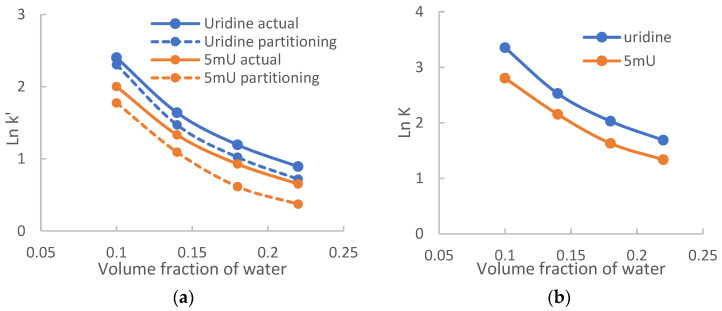
(**a**) The retention factors observed and calculated for partitioning for uridine and 5-methyluridine on TSKgel Amide-80 column in the mobile phase containing various levels of water at 20 mM ammonium acetate; (**b**) Distribution coefficients of uridine and 5-methyluridine.

The level of organic solvents (e.g., acetonitrile) in the mobile phase is critical in modulating both retention and selectivity in HILIC [28]. Both the partitioning (Equation (1)) and adsorption (Equation (2)) models predict a decrease in retention as the level of water in the mobile phase increases. However, it should be pointed out that the effects of acetonitrile or water on the partitioning and adsorption mechanisms are different. In hydrophilic partitioning, the distribution coefficients of polar compounds are correlated to the acetonitrile or water levels in the mobile phase; and the phase ratio is also influenced by the acetonitrile or water levels (Figure 2). To evaluate the effect of acetonitrile levels on hydrophilic partitioning, both the retention factors actually observed (*k*′) and calculated for partitioning ($k_{par}^{\prime }$) are evaluated for uridine and 5-methyluridine at 20 mM ammonium acetate since hydrophilic partitioning has been shown to be the predominant retention mechanism for both compounds. Figure 4a shows the logarithmic retention factors plotted against the volume fraction of water in the mobile phase. Both the actual retention factors (*k*′) and the calculated retention factors for partitioning ($k_{par}^{\prime }$) decrease with the volume fraction of water in a non-linear fashion.

The partitioning model (Equation (1)) predicts that logarithmic retention factors are linearly correlated to the volume fraction of water. However, two assumptions must be met for Equation (1) to be valid. First, the logarithmic distribution coefficient (Ln*K*) is linearly correlated to the volume fraction of water. Although linear relationship between Ln*k*′ and volume fraction of solvent has been demonstrated in RPLC, complex retention behaviors have been observed for some compound, which suggests that the logarithmic distribution coefficient may not be linearly correlated to the volume fraction of acetonitrile (stronger solvent) [21]. Second, the phase ratio needs to be constant as the volume fraction of water changes. At 20 mM ammonium acetate, the phase ratio is similar in the range of the volume fractions of water investigated in this study (Figure 2). Figure 4b shows the logarithmic distribution factors of uridine and 5-methyluridine plotted against the volume fraction of water. Ln*K* for both uridine and 5-methyluridine decreases with the volume fraction of water nonlinearly. Previous studies have demonstrated that the immobilized water layer has a complex structure consisting of a tightly bound water layer immediate to the surface of polar stationary phases and a diffuse layer with gradually increasing amount of acetonitrile [29,30,31]. The boundary between the mobile phase and the diffuse layer is not clearly defined [29,31]. In addition, the mobile phase and the adsorption water layer are not two immiscible solvents. This may explain the nonlinear relationship between the distribution coefficient and the volume fraction of water observed in Figure 4b. Comparing to Figure 4a, it is clear that the curves for the retention factors contributed by partitioning (dashed lines) follow the trend of Ln*K* in Figure 4b. Therefore, the observed retention behaviors in Figure 4a are more related to how the distribution coefficients are affected by the level of acetonitrile in the mobile phase. At lower ammonium acetate concentrations, the retention factors would also be affected by the difference in phase ratio at different acetonitrile levels.

#### 2.3.2. The Effect on Surface Adsorption

**Figure 5:**
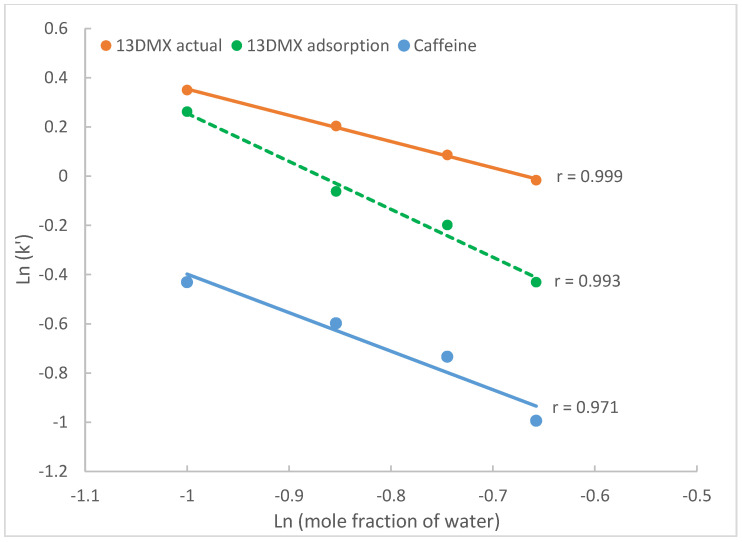
The actual retention factors of caffeine and 13DMX and the retention factors of theophylline attributed to adsorption on TSKgel Amide-80 column. The mobile phase contains 20 mM ammonium acetate.

In the adsorption driven process, solvent strength is a major factor influencing the interactions between the solutes and the ligands on the stationary phases [32]. Different volume fractions of water in the mobile phase change the mobile phase strength, potentially leading to a change in retention. To investigate the effect of acetonitrile content in the mobile phase, the retention factors of 1,3-dimethylxanthine and caffeine are evaluated since surface adsorption has been shown to be the predominant retention mechanisms for these compounds (Table 1). Figure 5 shows the logarithmic retention factors plotted against logarithmic mole fraction of water in the mobile phase containing 20 mM ammonium acetate. For caffeine, only the actual retention factors are plotted since partitioning is negligible. For theophylline (13DMX), both the actual retention factors (*k*′) and the calculated retention factors attributed to adsorption are ($k_{ads}^{\prime }$) are used for comparison. Linear regression was performed on the retention data of caffeine and 13DMX. Regression analysis reveals that first, the logarithmic retention factors attributed to adsorption (including caffeine) are linearly correlated to the logarithmic mole fraction of water, which is consistent with the adsorption model (Equation (2)); second, the logarithmic values of the actual 13DMX retention factors also show a linear relationship with the logarithmic mole fraction of water. This is most likely because surface adsorption contributes to most retention of 13DMX. However, the linear relationship between the actual retention factor and logarithmic mole fraction of water itself does not mean that surface adsorption is the only retention mechanism for 13DMX, even though the contribution of partitioning is small. In addition, both the actual retention factor of 13DMX and those attributed to adsorption decrease as the mole fraction of water increases as predicted by Equation (2); and the difference between the actual and adsorption-based retention factors becomes larger at higher water levels. This is because hydrophilic interaction contributes more to the observed retention in the mobile phase containing more water.

If the logarithmic retention factors of 13DMX and caffeine are plotted against the volume of fraction of water, linear lines would be observed. However, this should not be interpreted as the evidence to support that partitioning is the predominant retention mechanism for these compounds according to Equation (1). Without quantitative information on the contribution of each retention mechanism, it would be difficult to reach a reliable conclusion.

#### 2.3.3. The Effect on Combined Hydrophilic Partitioning and Surface Adsorption

**Figure 6:**
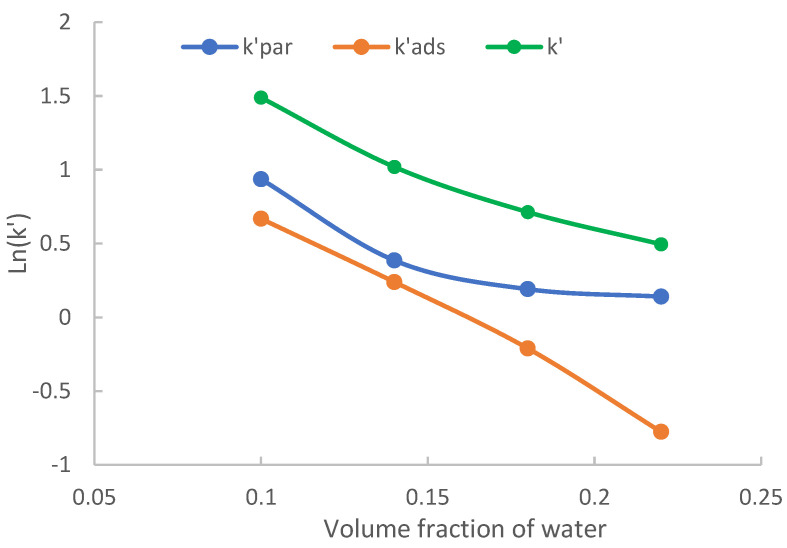
The actual retention factors (*k*′), the retention factors attributed to hydrophilic partitioning ($k_{par}^{\prime }$) and surface adsorption ($k_{ads}^{\prime }$) of 3-methylxanthine on TSKgel Amide-80 column. The mobile phase contains 20 mM ammonium acetate.

Table 1 shows that both hydrophilic partitioning and surface adsorption are significant retention mechanisms for 3-methylxanthine. Therefore, the level of acetonitrile in the mobile phase is expected to have a significant effect on the retention of 3-methylxantine through both hydrophilic partitioning and surface adsorption. Figure 6 shows logarithmic values of the actual retention factor (*k*′) and retention factors attributed to partitioning ($k_{par}^{\prime }$) and adsorption ($k_{ads}^{\prime }$) obtained on the polyacrylamide phase in the mobile phase containing 20 mM ammonium acetate at various acetonitrile levels. As shown in Figure 6, the actual retention factor (*k*′) decreases with increasing volume fraction of water in a non-linear fashion. The retention factors attributed to hydrophilic partitioning also decrease in a trend that largely parallels the distribution coefficients, similar to what is observed for uridine and 5-methyluridine (Figure 4). In comparison, the retention factors attributed to surface adsorption decrease in a different pattern; and the decrease is more significant at larger volume fractions of water. 3-Methylxanthine provides an example that the plot of Ln*k*′ vs. volume fraction of water or logarithmic mole fraction of water does not fit either the partitioning model (Equation (1)) or adsorption model (Equation (2)). Without quantitating the contributions of the partitioning and adsorption mechanisms, it would be impossible to correctly understand the retention mechanism. The decrease in retention at higher water levels is more related to the decrease in the retention factor attributed to surface adsorption than hydrophilic partitioning. This result highlights again the importance of quantitative retention assessment so the retention mechanisms can be correctly understood.

## 3. Materials and Methods

### 3.1. Materials and Reagents

HPLC grade acetonitrile (CAN) was purchased from Sigma-Aldrich (St. Lous, MO, USA). Water was obtained from an in-house Milli-Q water purification system (Millipore, Bedford, USA). Toluene, uridine, 5-methyluridine, caffeine, 3-methylxanthine (3MX) and theophylline (1,3-dimethylxanthine, 13DMX) were all acquired from Fisher Scientific (Hampton, NH, USA). Ammonium acetate (ultra-pure) was purchased from Amresco Inc. (Solon, OH, USA). A toluene solution was prepared by adding 20 μL toluene to 1 mL acetonitrile. Individual test compound solutions were prepared by dissolving 10 mg of each compound in 10 mL acetonitrile and water mixture (85/15, *v*/*v*). The test solution was prepared by mixing individual test compound solutions at equal volumes and adding 20 μL toluene solution.

TSKgel Amide-80 column (3 μm particle size, 4.6 mm ID and 15 cm long) was purchased from Tosoh Bioscience (King of Prussia, PA, USA). Epic HILIC column (5 μm particle size, 4.6 mm ID and 25 cm long) was obtained from ES Industry (West Berlin, NJ, USA). YMC PVA column (5 μm particle size, 4.6 mm ID and 25 cm long) was from YMC America Inc. (Devens, MA, USA).

A stock solution of ammonium acetate (200 mM) was prepared by dissolving an appropriate quantity in purified water. The pH of the stock solution was around 6.8 and no additional pH adjustment was made. The mobile phase was mixed acetonitrile, water and ammonium acetate stock solution at propriate proportions to achieve desired acetonitrile level and ammonium acetate concentration online by quaternary gradient pumps.

### 3.2. Equipment and Methods

An Agilent 1260 HPLC system (Palo Alto, CA, USA) equipped with an online vacuum degasser, a quaternary gradient pump, an autosampler, a themostatted column compartment, a variable UV detector was used for all the experiments. Retention data was collected and processed by ChemStation for LC and LC/MS (Rev. C.01.06). All experiments were performed isocratically at the flow rate of 1.0 mL/min. The column temperature was set at 25 °C. The test compounds were detected by UV at 250 nm and the injection volume was 2 μL.

## 4. Conclusions

The retention mechanisms of five selected test compounds were evaluated by quantitatively determining the contributions of hydrophilic partitioning and surface adsorption to the observed retention in HILIC. Quantitative assessments reveal that hydrophilic partitioning and surface adsorption represent two ends of a mechanism spectrum for non-ionized polar compounds. The retention of polar compounds may be controlled by hydrophilic interaction, surface adsorption or a combination of both. For example, the retention of uridine and 5-methyuridine is predominantly controlled by partitioning; and surface adsorption is the predominant retention mechanism for caffeine and theophylline. In comparison, 3-methylxanthine is retained by both the partitioning and adsorption mechanisms. Therefore, the retention mechanisms in HILIC are very complex depending on the characteristics of polar compounds. In addition, the analysis of quantitative retention data of uridine and 5-methyluridine suggests that the adsorbed water layer on the polyhydroxy stationary phase might not be completely accessible to polar compounds in the partitioning process. This study is the first report of this phenomenon and further investigation is warranted to gain a better understanding.

Quantitative assessments of the retention mechanisms provide more insights into the effect of acetonitrile on retention in HILIC. The attempts to explain the retention behaviors of polar compounds simply using either the partitioning model (Equation (1)) or adsorption model (Equation (2)) is presumptive or may even lead to erroneous conclusions. It is often not clearly stated in literature that the partitioning model (Equation (1)) requires two assumptions, existence of a linear relationship of Ln*K* vs. volume fraction of water and a constant phase ratio. The results of this study demonstrate that these assumptions may not be valid in HILIC. For the compounds primarily controlled by hydrophilic partitioning, stronger retention at higher acetonitrile levels is mainly due to larger distribution coefficients. Quantitative assessments allow the determination of the retention attributed to surface adsorption, which is shown to follow the adsorption model (Equation (2)). For the compounds controlled by both partitioning and adsorption, stronger retention at higher acetonitrile levels is attributed to both larger distribution coefficients and stronger surface adsorption. The current study only investigated the retention of five selected compounds on the TSKgel Amide-80 column and two polyhydroxy phases (Epic HILIC and YMC PVA columns). Obviously, more studies are needed to evaluate the retention mechanisms of a wide variety of compounds (e.g., carbohydrates, small organic acids, and polar pharmaceutical compounds). In addition, more stationary phases with diverse ligand structures will be investigated in future studies. Nevertheless, this study clearly demonstrates that quantitative determination of retention contributions makes it possible to unambiguously assign retention mechanisms in HILIC.

## Figures and Tables

**Table 1:** Distribution coefficients and retention factors contributed by partitioning and adsorption for five selected test compounds on TSKgel Amide-80 column at 25 °C ^1^.

Test Compound	Distribution Coefficient (*K*)	Retention Factor by Partitioning ($k_{par}^{\prime }$)	Retention Factor by Adsorption ($k_{ads}^{\prime }$)
Uridine	28.6	10.0 (90.9%) ^2^	0.74 (6.7%)
5-methyluridine	16.5	5.90 (79.7%)	1.47 (19.8%)
3-methylxanthine	6.9	2.55 (57.6%)	1.95 (44.1%)
1,3-methylxanthine	0.7	0.36 (22.9%)	1.30 (83.2%)
Caffeine	NA	NA	0.54 ^3^

^1^ Mobile phase: acetonitrile/20 mM ammonium acetate (90/10, *v*/*v*). ^2^ Numbers in parenthesis indicate the percentage of $k_{par}^{\prime }$ or $k_{ads}^{\prime }$) in the observed retention factor. ^3^ Average of the actual retention factors at 4, 12, and 20 mM ammonium acetate (90% acetonitrile).

**Table 2:** Phase ratios of two polyhydroxy stationary phases and distribution coefficients and intercepts of uridine and 5-methyluridine ^1^.

	Columns	Phase Ratio (∅)	Distribution Coefficient (*K*)	Intercept	K∅
Uridine	YMC PVA	0.205	8.76	−0.22	1.80 (116%) ^2^
	EPIC HILIC	0.245	8.65	−0.30	2.12 (116%)
5-methyl uridine	YMC PVA	0.205	6.17	0.01	1.26 (101%)
	EPIC HILIC	0.245	6.08	0.00	1.49 (100%)

^1^ Mobile phase: acetonitrile/20 mM ammonium acetate (85/15, *v*/*v*). ^2^ Numbers in parenthesis indicates the percentage of *K*∅ in the observed retention factor.

## Data Availability

The data is already presented in the manuscript.

## Sample Availability

Samples of the compounds are available from the authors.
